# Pulmonary Pharmacokinetics of Antibody and Antibody Fragments Following Systemic and Local Administration in Mice

**DOI:** 10.3390/pharmaceutics16101259

**Published:** 2024-09-27

**Authors:** Prabhas Jagdale, Ashwni Verma, Dhaval K. Shah

**Affiliations:** Department of Pharmaceutical Sciences, School of Pharmacy and Pharmaceutical Sciences, The State University of New York at Buffalo, Buffalo, NY 14214, USA; prabhasb@buffalo.edu (P.J.); ashwnive@buffalo.edu (A.V.)

**Keywords:** pharmacokinetics, pulmonary, lungs, intratracheal instillation, bronchoalveolar lavage, antibody, antibody fragments, protein therapeutics

## Abstract

**Objective:** This study aimed to investigate the effect of molecular size on the pulmonary pharmacokinetics (PK) of proteins following systemic and local administration in wild-type mice. **Methods:** A non-cross-reactive antibody trastuzumab, and F(ab′)2, Fab, and scFv fragments of this antibody were used for the investigation. Proteins were injected intravenously or via intratracheal instillation, and PK was measured in plasma, lungs, trachea, bronchi, and bronchoalveolar lavage (BAL) using ELISA. Concentrations in BAL were urea normalized. **Results:** Following systemic administration, the biodistribution coefficient (BC) for lungs, trachea, bronchi, and BAL was 11%, 11%, 15%, and 2% for the antibody; 15%, 7%, 13%, and 8% for F(ab′)2; 25%, 17%, 28%, and 46% for Fab; and 14%, 1%, 2%, and 50% for scFv. The antibody exposure in BAL was ~50-fold lower than plasma and ~5–7-fold lower than lung tissues. A tissue-dependent BC vs. molecular size relationship was observed, where distribution in tissues was the highest for Fab (50 kDa), and scFv demonstrated the highest distribution in the BAL. PK data generated following local administration were quite variable; however, local dosing resulted in BAL exposures that were 10–100-fold higher than those achieved after systemic dosing for all proteins. The BAL antibody concentrations were 100–1000-fold higher than plasma concentrations initially, which normalized by day 14. For most proteins, local dosing resulted in higher lung concentrations than trachea and bronchi, opposite to what was observed after systemic dosing. **Conclusions:** The PK data presented here provide an unprecedented quantitative insight into the effect of molecular size on the pulmonary disposition of proteins following systemic and local administration.

## 1. Introduction

The use of protein therapeutics has been rising steadily over the past few decades, with more than 240 different therapeutic proteins and peptide drugs approved by the Food and Drug Administration (FDA) since the 1980s [1]. These drugs are unique because they have high specificity to the target and are involved in highly complex pathways that small-molecule drugs cannot mimic. As a result of high specificity and affinity to the target, they do not interfere with other biological processes, thus reducing the risk of off-target toxicity [2]. Since the introduction of the first recombinant protein, human insulin [3], proteins have been explored to elicit varied pharmacological actions. Today, therapeutic proteins are used as (1) replacement proteins; (2) proteins that augment a pathway; or (3) proteins that provide a novel function, (4) interfere with mechanism or organism, and (5) deliver payloads to specific targets [3]. Additionally, despite the risks associated with the development of immunogenicity [4], protein therapeutics remain a significant class of drugs explored in different disease conditions and settings.

Over the years, there has been a rise in efforts to explore the use of proteins for treating pulmonary disorders [5] such as chronic obstructive pulmonary disease (COPD), cystic fibrosis, asthma, coronavirus disease 19 (COVID-19), bacterial infections, and other pulmonary diseases [6]. About six monoclonal antibodies (mAbs) have received emergency use authorization for managing the COVID-19 virus, and many more biologics have been explored for managing COVID-19 therapeutically [7]. MAbs also have potential as antibacterial drugs targeting toxins or bacterial surface proteins. The FDA has approved three mAbs targeted against bacteria, and many more are in development; some of these mAbs target lung infections like pneumonia and inhalation anthrax [8].

Although significant efforts are underway to develop protein therapeutics to treat pulmonary disorders and lung infections, there is a need for a better understanding of the PK of these molecules in different regions of the lungs. Considering the pharmacodynamics of proteins is closely related to their PK, more efforts should be made to better understand the disposition of proteins in different regions of the pulmonary tissues. Many pulmonary disorders like COPD, airway inflammation, interstitial lung diseases, and damage from bacterial toxins lead to changes in the composition of the epithelial lining fluid (ELF) [9,10], and high ELF concentrations of therapeutic proteins are desirable to manage such conditions. Hence, it is crucial to investigate the penetration of proteins into the lung ELF.

The delivery of proteins to the lungs can be achieved through systemic or local (e.g., inhalation) routes. The inhalation route has also been explored to deliver biologics systemically apart from targeting the lungs [11]. Although several in vivo studies have been published that look into the penetration of mAb and its fragments into lung tissue as a whole [12,13,14] and the penetration of mAb and other therapeutic proteins into plasma after pulmonary dosing [14,15], no effort has been made to study the pulmonary disposition of protein therapeutics comprehensively. The molecular size of proteins is a factor that has been reported to affect the PK of drugs in the alveolar epithelial space [16,17,18,19]. Studies have shown that smaller-sized proteins can be absorbed faster and to a greater extent than larger ones after pulmonary administration. It has also been reported that smaller-sized proteins can penetrate quicker and accomplish higher levels in the lungs than larger-sized proteins after systemic administration [18]. However, the systemic delivery of proteins for targeting the lungs involves a delay before proteins reach the epithelial lining fluids in the lungs. In addition, the fact that, after systemic administration, only a fraction of plasma concentration reaches the lungs [18] might make inhalation a preferred route for delivering drugs to the lungs [19,20]. Thus, it remains to be seen if pulmonary administration provides a significant benefit over systemic administration for achieving high local concentrations of protein therapeutics in the lungs.

**Figure 1:**
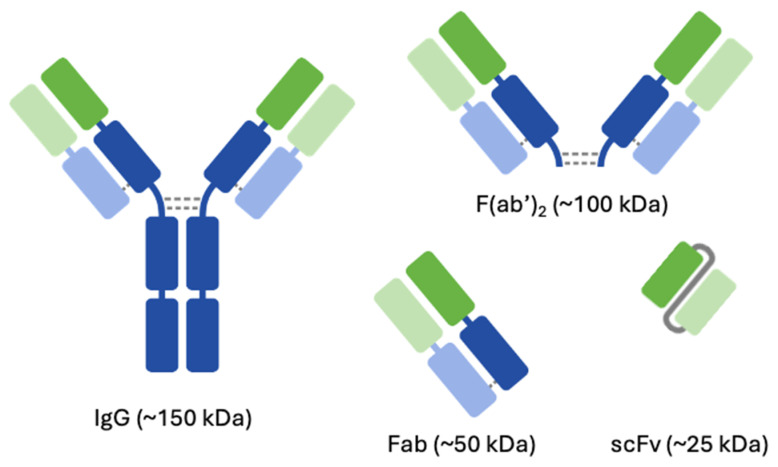
Schematic representation of IgG, F(ab′)2, Fab, and scFv fragments used as tool molecules to study the pulmonary disposition of proteins.

In the present study, we addressed this knowledge gap by conducting an in vivo biodistribution study to investigate the disposition of trastuzumab, as well as its fragments, namely F(ab′)2 (two fragment antigen-binding regions of an antibody), Fab (fragment antigen binding of an antibody), and scFv (single-chain fragment variable region of an antibody) (Figure 1), in mice after intravenous and pulmonary dosing. Trastuzumab is a recombinant humanized IgG1 (hereafter called IgG) mAb that binds to human epidermal growth factor 2 (HER2). It is approved for HER2 overexpressing breast cancer and metastatic gastric or gastroesophageal junction adenocarcinoma. It is dosed through intravenous infusion and acts by blocking HER2 signaling and, primarily, by killing HER2-expressing cells through antibody-dependent cellular cytotoxicity (ADCC) [21]. F(ab′)2, Fab), and scFv fragments of trastuzumab can also bind to HER2 and elicit some pharmacology by blocking HER2 signaling, but due to the absence of the Fc region, they cannot induce ADCC. All the proteins used in our study bind HER2 and are non-cross-reactive in mice, thus making them ideal proteins for understanding the disposition of proteins. We quantified the penetration of different-sized proteins in different pulmonary tissues: the lungs, upper airway, lower airway, and BAL samples. The local delivery of proteins was achieved via non-invasive intratracheal administration. After systemic administration, we explored the relationship between molecular size and the penetration of proteins in different pulmonary tissues. As such, the data presented here give an insight into the pulmonary PK of proteins and the rate and extent of their exposure in various regions of the lungs following systemic and local administration.

## 2. Materials and Methods

### 2.1. Material

Herceptin^®^ (Trastuzumab) was purchased from a local hospital in lyophilized form and reconstituted to 20 mg/mL using sterile Milli Q water (MilliporeSigma™, Burlington, MA, USA) as provided in the instruction sheet. Capture and detection antibodies for ELISA were purchased from Bethyle laboratory (Montgomery, TX, USA). Human Her-2 was purchased from Sino Biologicals (Beijing, China). ELISA plates, PNPP, and TMB reagents were purchased from Thermo Scientific (Waltham, MA, USA).

### 2.2. Development and Characterization of Different-Sized Proteins

Reconstituted trastuzumab has some excipients for improved stability such as L-lysine, a,a-trehalose dihydrate, and Tween 20. These excipients were removed using Amicon^®^ ultracentrifugal filters 50 kDa, filter (MilliporeSigma™, Burlington, MA, USA) before further analyses like quantification, digestion, or chemical conjugation. The other proteins (Figure 1), namely F(ab′)2 (100 kDa), Fab (50 kDa), and scFv (27 kDa) fragments of trastuzumab (150 kDa), were generated in-house. SDS-PAGE analysis (Bio-Rad, Hercules, CA, USA) confirmed the molecular size and purity of developed antibody fragments.

#### 2.2.1. Preparation of Fab Fragment of Trastuzumab

The Fab fragment of trastuzumab was generated in the lab by selective digestion of antibody using Pierce™ Fab Preparation Kit (Thermo Scientific™) that cleaves antibody above the hinge region and makes two identical Fab and one Fc from a single antibody. The cleaved Fab from the antibody was purified using a hydroxyapatite column (CHT^TM^, Bio-Rad^®^). Briefly, 4 mg of trastuzumab was purified using the Zeba Spin Desalting Column (Thermo Scientific™) by centrifugation at 1000× *g* for 2 min. This step was repeated three times. The purified trastuzumab was incubated with 0.125 mL of papain-immobilized resin in 0.5 mL of digestion Buffer provided with Pierce™ Fab Preparation Kit (Thermo Scientific™) at 37 °C for 12 h in a shaking incubator. After digestion, the product was centrifuged in the provided centrifuge column at 5000× *g* for 1 min, where papain-linked beads were trapped in the column and dissolved digested antibodies were collected. The mixture was then purified using the hydroxyapatite column (CHT^TM^, Bio-Rad^®^). Chromatographic separation was performed using mobile phage composed of solution A (10 mM sodium phosphate with 5 ppm calcium chloride, pH 6.5) and solution B (500 mM sodium phosphate with 5 ppm calcium chloride, pH 6.5). The optimal chromatographic separation between Fab2, FC, and undigested Ab was achieved by changing the gradient of the two solutions. Further, the identity of each peak from the chromatogram was determined by performing SDS-PAGE. Before the experiments, the final product was concentrated and buffer-exchanged in phosphate-buffered saline (PBS).

#### 2.2.2. Preparation of F(ab′)2 Fragment of Trastuzumab

Similar to Fab generation, the F(ab′)2 fragment of trastuzumab was generated using a Pierce™ F(ab′)2 Preparation Kit (Thermo Scientific™) that cleaves antibodies below the hinge region and generates one F(ab′)2 and one Fc fragment from a single antibody. Briefly, the purified trastuzumab was incubated with 0.125 mL of pepsin-immobilized resin in 0.5 mL of digestion buffer (provided with the Pierce™ F(ab′)2 Preparation Kit (Thermo Scientific™)) at 37 °C in a shaking incubator for 8 h. After digestion, the product was centrifuged in the provided centrifuge column at 5000× *g* for 1 min, where pepsin-linked beads were trapped in the column and dissolved the digested antibodies, and a minute amount of full-length Abs was used as the eluent. The obtained eluent was further purified using a hydroxyapatite column as described in the Fab preparation protocol.

#### 2.2.3. Preparation of scFv Fragment of Trastuzumab

CHO cells producing trastuzumab scFv were developed as reported in our previous publication [22]. Briefly, scFv plasmid was designed by linking the VH and VL sequence using a flexible polyglycine liker (GGGGS)3. Two restriction enzyme sites for Nhel and BamHI were included at the sequence’s beginning and end, respectively. After the plasmid was synthesized by Genscript^®^ (Piscataway, NJ, USA), *scFv gene* cloning in the FRT plasmid was performed by ligating digested *scFv gene* product in the FRT vector (pcDNA™5/FRT) using T4 ligase. The developed FRT-scFv plasmid was transfected in *E. coli*, and the transfected colonies were selected using Amp antibiotic resistance on the Agar plate. The FRT-scFv plasmid was extracted from the grown colonies, and the sequence of the inserted scFv fragment was confirmed by Sanger sequencing using primer for the T7 prompter on the FRT plasmid. After confirming the inserted sequence, the FRT-scFv plasmid was transfected in CHO cells along with Pog 44 using a previously optimized plasmid ratio of 1:9 (FRT-scFv: Pog44). The transfected CHO cells were selected using 1 mg/mL hygromycin-B in CD-CHO media. The transfected CHO cells were then sub-cloned in 96-well plates for two rounds to separate monoclones. The supernatant of each well was collected and analyzed by ELISA to determine high-producing clones. The best-obtained clones were amplified in T-75 flasks using CD-CHO media and cultured as a suspension in 100 mL and 1 L flasks on a shaking incubator. When culture media became highly confluent (10^7^ cells/mL with about 90% cell viability), culture media were centrifuged at 10,000 rpm for 10 min, and the supernatant was collected and filtered using a 0.22 µm filter. scFv was purified from the filtered culture media using the His Gravitrap column (His GraviTrap™ Cytiva, Marlborough, MA, USA). Bound scFv to the column was eluted using 2 column volumes of 500 mM imidazole in phosphate buffer (pH 6.0). The eluted scFv with imidazole was buffer-exchanged 3 times in PBS using 10 kDa cutoff Amicon centrifuge tubes (Millipore Sigma Amicon^®^ Ultra Centrifugal Filter, 10 kDa MWCO, Catalog No—UFC9010). Further, the obtained scFv was verified by performing an SDS-PAGE analysis.

### 2.3. Pulmonary Administration of Protein Therapeutics

The local delivery of proteins in the lungs was accomplished via intratracheal instillation using a catheter. First, the animals were anesthetized through the inhalation of isoflurane and suspended using their maxillary incisors on the intubation platform at a 90° angle to the ground. The tongue was pulled out gently using blunt-tipped forceps to clear the air passage while holding the tongue outside with the non-dominant hand. The catheter was then inserted in the trachea in line with the central axis of the animal [23]. The correct placement of the catheter into the trachea was confirmed by connecting the catheter to an IV drip line and looking for condensation in the drip line due to breathing or by adding 10 µL of PBS to the catheter to check for pulses in PBS due to animal breathing (Figure S1). To evaluate the efficacy of administration via the pulmonary route, we performed experiments with dye (Trypan blue). We analyzed the presence of dye in the lungs and GI tract to understand the delivery efficiency through pulmonary dosing. Several attempts were made to accurately administer the dose to the lung airway while avoiding its entry into the esophagus and stomach. The anesthetized animals were administered with varying amounts of dye (50 to 100 µL of 100 times diluted Trypan blue in PBS) using intratracheal instillation. After administration, animals were dissected, the lung and GI tract were isolated, and further visual examination was performed to detect the presence of Trypan blue, as shown in Figure S2. The breathing and movement of the animals were continuously monitored during the entire process. Once the intubation was confirmed, the proteins were dosed into the trachea by connecting the catheter to a syringe and instilling 100 µL of protein sample. The animals were then put back into their cages and kept under heat until they recovered entirely from anesthesia.

### 2.4. In Vivo PK Study to Investigate the Pulmonary Disposition of Protein Therapeutics

**Figure 2:**
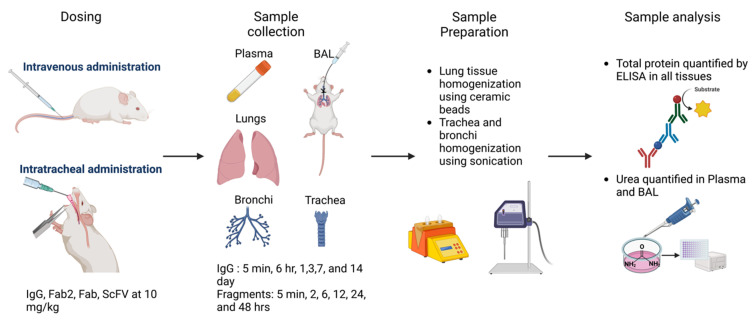
Graphical representation of the study design used to determine the PK of different-sized proteins in pulmonary tissues. C57BL/6J mice were dosed with trastuzumab and trastuzumab fragments at 10 mg/kg dose level. Animals were sacrificed at predetermined time points, and plasma, lungs, trachea, bronchi, and BAL fluid were collected. Samples were prepared and analyzed using ELISA. Urea assay was performed on plasma and BAL samples to correct for dilutions in BAL fluid.

Wild-type male C57BL/6J mice were used to perform all the in vivo PK experiments. All the experiments were performed as per IACUC protocol (PROTO202000054). As shown in the study design in Figure 2, all the proteins were administered at 10 mg/kg dose via 50 microliter injection intravenously or 100 microliter intratracheally to facilitate high enough concentrations for measurement. A total of 150 mice were used for this investigation; 6 animals were used as controls, 72 were given the proteins systemically, and 72 were administered the drug intratracheally. Each group of 72 mice was further divided into four groups of 18 animals, administered with one of the four proteins. Three mice from each group were sacrificed at six different time points. For trastuzumab, animals were sacrificed at 5 min, 6 h, 24 h, 3 days, 7 days, and 14 days. For all the fragments of trastuzumab, the time points were 5 min, 2 h, 6 h, 12 h, 24 h, and 48 h. At the time of sacrifice, a blood sample, BAL fluid (collected based on the published protocol [24]), lungs, bronchi, and trachea were collected for measuring protein concentrations via ELISA.

The systemic delivery of proteins was carried out via penile vein injections; each animal was anesthetized through the inhalation of isoflurane in a closed chamber. When the mouse lost its paw reflex and did not respond to a foot pinch, the anesthetized mouse was placed in a nose cone connected to an isofluorane vaporizer to maintain anesthesia. The mouse was maintained on gaseous anesthesia for the dosing period. About 5% isoflurane was used for induction, and 2–3% was used for maintenance. Post-dosing, the animals were kept on a heating pad and monitored during recovery from anesthesia. The local delivery of proteins was performed via intratracheal instillation, as described above.

As mentioned previously, animals were sacrificed at predetermined time points. The animals were given a lethal dose (300 mg/kg) of FATAL-PLUS (Vortech Pharmaceuticals, Ltd., Dearborn, MI, USA) through an IP injection. Once the animal was confirmed dead, blood was collected through cardiac puncture. For bronchoalveolar lavage, a small incision was made in the trachea; a catheter was introduced through this incision and was held in place by tying a suture. One thousand microliters of PBS was injected into the lungs, followed by aspiration while gently massaging the thorax. The lung lavage was then collected, followed by the collection of lungs, trachea, and bronchi tissues. The tissues were cleaned of residual blood by dabbing onto a tissue and then transferred and stored in a −80 °C freezer.

### 2.5. Tissue Homogenization

Lung, trachea, and bronchi samples were weighed and homogenized in a 1:4 *w*/*v* Pierce^®^ RIPA Buffer (Thermo Fisher Scientific) containing 1 × HaltTM Protease Inhibitor Cocktail (Thermo Fisher Scientific). Lung tissue was homogenized using a BeadBugTM microtube homogenizer (Benchmark, Ames, IA, USA). Each homogenization tube contained seven zirconium beads (3.0 mm, Benchmark) along with lung tissue and RIPA buffer; tubes were homogenized for 15 s, followed by 30 s ice cool down, repeated three times [13]. Due to the small tissue volumes of the trachea and bronchi, these samples were homogenized using a FisherbrandTM Model 120 Sonic Dismembrator (Fisher Scientific) with a 1/8″ probe. Each tissue was subjected to three cycles of 3 s on and 5 s off sonication, with the amplitude set to 45% [25].

All the homogenized tissue samples were then incubated on ice for 2 h to establish equilibrium. The tissue samples and BAL samples were centrifuged at 15,000× *g* for 15 min at 4 °C; the supernatant was then collected and analyzed using ELISA.

### 2.6. Analytical Method Development

Enzyme-linked immunosorbent assay (ELISA) was used to quantify the concentrations of proteins in tissues. The sandwich ELISA method used for quantification was as follows: (a) coating a 384-well plate with capture antibody and incubating the plate at 4 °C overnight; (b) blocking non-specific binding sites by adding 10% BSA blocking buffer, followed by a 1 h incubation at room temperature; (c) adding samples, standards, and QCs to the plate followed by a 2 h incubation at room temperature; and (d) adding detection antibody to the plate, incubating for 1 h at RT, and adding the substrate of the plate, followed by a kinetic read for 45 min. Between each step, the plate was washed three times with 1% PBS–Tween (0.05% Tween-20 in 1× PBS, no pH adjustment), followed by three washes with distilled water. For trastuzumab, the capture antibody used was anti-human IgG-Fc, F(ab′)2 was captured with anti-human IgG-F(ab′)2, and for Fab and scFv fragments of trastuzumab, Her2 was used as capture protein. Briefly, 30 µL of capture proteins at 5 µg/mL concentration in 20 mM Na2HPO4 was added to each well. Plates were blocked with 90 µL of blocking buffer, and 30 µL volumes of samples, standards, and QCs were added to respective wells in triplicates and incubated on a plate shaker. Thirty microliters per well of the 1.4 ng/mL of goat anti-human IgG-F(ab′)2 cross-adsorbed F(ab′)2conjugated with alkaline phosphatase in washing buffer was used as the secondary antibody for IgG, Fab, and F(ab′)2. In contrast, an anti-6X HIS tag antibody (HRP-conjugated) was used to detect scFv. Thirty microliters per well of p-nitrophenyl phosphate solution (1 mg/mL in 1× diethanolamine substrate buffer) was used as the coloring agent for IgG, Fab, and F(ab′)2, while 3,3′,5,5′-tetramethylbenzidine substrate solution was used with scFv. The change in absorbance was measured over time (dA/dt) at 405 nm for 45 min using a Filter Max F5 microplate analyzer (Molecular Devices, Sunnyvale, CA, USA). All standard curves were fitted using a 5-parameter logistic equation.

Three different Elisa assays were performed for each protein, with different tissue matrices for plasma, lung, and BAL. Plasma samples were diluted 1000-fold for trastuzumab, while for F(ab′)2, Fab, and scFv, the samples were diluted 300-fold. Tissue samples from the lungs, trachea, and bronchi were diluted 100-fold in the lung tissue matrix, and BAL samples were diluted 5-fold in the blank BAL matrix.

### 2.7. Urea Assay

The protein concentrations in the BAL samples were normalized by urea. While isolating BAL, the epithelial lining fluid was diluted in PBS, and this dilution factor varied anywhere between 30- and 70-fold. Hence, we normalized all the BAL data with plasma urea levels to account for the dilution. A commercially available colorimetric urea assay kit (LS Bio LS-K331) was used.

The urea assay method used for quantifying urea concentrations was as follows: (a) for plasma samples, 5 µL of sample along with blank (DI water) and standard (urea concentration 5 mg/dL) were added to a 96-well plate in duplicates; (b) for BAL sample, 50 µL of samples, with 50 µL of blank and 50 µL of standards (50 mg/dL) were added to a 96-well plate in duplicates; (c) the working reagent was prepared by adding equal volumes of reagent A and reagent B, and 200 µL of the freshly prepared working reagent was added to the samples; (d) the plate was lightly tapped to mix the samples and reagents and incubated for 20 min for plasma and 50 min for BAL samples at room temperature; and (e) the absorbance was measured at 520 nm.

The urea concentration of the sample was calculated as follows:

$$\left[Urea\right]=\frac{OD_{Sample}-OD_{Blank}}{OD_{Standard}-OD_{Blank}}\times DF\times Standard$$

where *OD* is the optical density (*OD*) at 520 nm wavelength, and *DF* is the dilution factor of the sample.

### 2.8. Data Analysis

The PK parameters, the area under the curve (*AUC*_0–*last*_), maximum concentration (*Cmax*), elimination half-life (*T1/2*), and time to *Cmax* (*Tmax*) were calculated using non-compartmental analysis in MATLAB/Simbiology R2019b [26]. Tissue Biodistribution coefficient (*BC*%) was calculated as the ratio of exposure (*AUC*_0–*last*_) of protein in tissue to plasma as follows:

$$BC{\%}_{tissue}=\frac{AUC_{Tissue_{\left(0–last\right)}}}{AUC_{Plasma_{\left(0–last\right)}}}\times 100$$

BAL-to-lung penetration was calculated as the ratio of exposure of protein in BAL to lungs (*AUC*_0–*last*_). The dose-normalized PK was calculated by normalizing the concentration of proteins in each tissue at every time point to the moles of protein dosed to the animal.

## 3. Results

### 3.1. Production and Characterization of Antibody Fragments

The antibody fragments of different molecular sizes were developed by performing digestion per the instructions provided with the papain and pepsin digestion kits. After purification, the binding and purity of the developed antibody fragments were evaluated by performing ELISA and SDS-PAGE analysis. SDS-PAGE analysis showed >95% purity with a significant single band (Figure S3).

### 3.2. Development of ELISA and Urea Assay for Quantifying Protein Levels

Figure S4 shows the ELISA calibration curve for trastuzumab, as well as F(ab′)2, Fab, and scFv fragments of trastuzumab, in the lung tissue matrix. Standard curves utilizing the five-parameter equation had R^2^ values of ≥0.95 in the SoftMax^®^ Pro software, Molecular Devices, San Jose, CA, USA. Elisa allowed for the quantification of IgG, F(ab′)2, Fab, and scFv in lung tissues with a lower limit of quantification (LOQ) at 100 ng/mL for IgG and F(ab′)2 and 50 ng/mL for Fab and scFv. The lower limit of quantification (LOQ) for BAL samples was 30–60 ng/mL, depending on the urea dilution factor. The LOQ for the plasma level of proteins was 100 ng/mL.

The standard curve for urea assays is shown in Figure S5. In the urea concentration range of 0–100 mg/dL, there was a linear relation between absorbance at 520 nm and urea concentration. Each sample was run in duplicate. For low-urea samples like BAL, the incubation time was extended to 50 min instead of 20 min for the plasma samples.

### 3.3. In Vivo PK Study

#### 3.3.1. Trastuzumab PK

**Figure 3:**
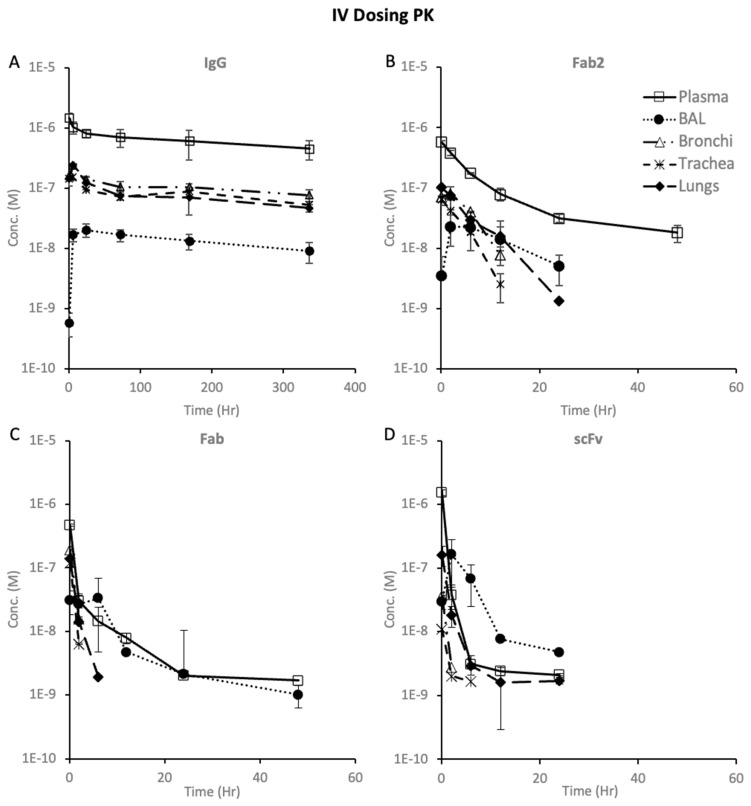
Pulmonary PK (mean ± S.D. (*n* = 3)) after systemic dosing of proteins at 10 mg/kg dose: (**A**) trastuzumab IgG, (**B**) trastuzumab F(ab′)2 fragment, (**C**) trastuzumab Fab fragment, and (**D**) trastuzumab scFv fragment.

The PK of trastuzumab in plasma, BAL, lungs, trachea, and bronchi after systemic administration of 10 mg/kg dose is presented in Figure 3A. The plasma *T1*/*2* of trastuzumab was found to be 17.3 days, which is on the higher end of the reported half-lives for the antibody in mice but still in line with what has been reported in the literature for trastuzumab [27]. The *Cmax* in the lungs, trachea, and bronchi was at 6 h, while the *Cmax* in BAL was at 24 h, which shows a significant delay in reaching a maximum concentration in BAL. Trastuzumab concentrations were slightly higher in the bronchi than in the trachea and lungs. The exposure in BAL was about 50-fold lower than in the plasma, suggesting that a very small percentage of the drug from the systemic circulation reached the epithelial lining fluid. The exposure of IgG in BAL was about 5–7-fold lower than in the lungs, trachea, and bronchi (Table 1).

**Figure 4:**
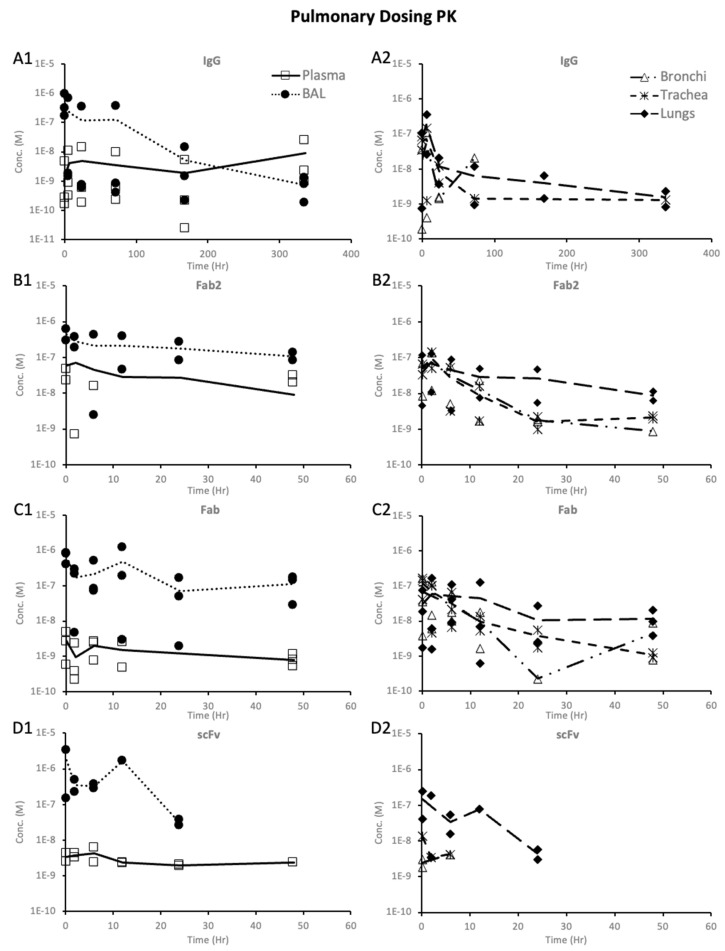
Trastuzumab protein PK in pulmonary tissues after intratracheal administration of 10 mg/kg dose: (**A**) trastuzumab IgG ((**A1**)—plasma and BAL, (**A2**)—bronchi, trachea, and lungs); (**B**) trastuzumab F(ab′)2 ((**B1**)—plasma and BAL, (**B2**)—bronchi, trachea, and lungs); (**C**) trastuzumab Fab ((**C1**)—plasma and BAL, (**C2**)—bronchi, trachea, and lungs); and (**D**) trastuzumab scFv ((**D1**)—plasma and BAL, (**D2**)—bronchi, trachea, and lungs). Solid lines represent mean concentration, and symbols represent individual animal concentration.

**Figure 5:**
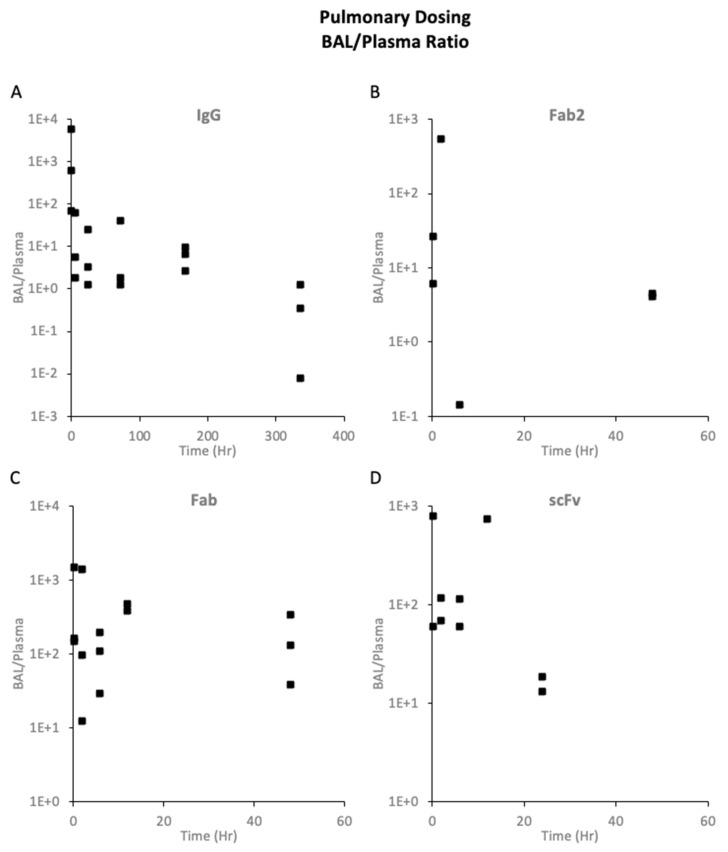
BAL-to-plasma ratio for proteins after 10 mg/kg local dosing: (**A**) trastuzumab IgG, (**B**) trastuzumab F(ab′)2 fragment, (**C**) trastuzumab Fab fragment, and (**D**) trastuzumab scFv fragment. Each symbol represents the BAL-to-plasma ratio of an individual animal.

The pulmonary PK of trastuzumab after pulmonary dosing is shown in Figure 4(A1,A2). There was significant inter-individual variability in the PK data, possibly because of the variability in the dosing of the proteins. Figure 4(A1) shows the trastuzumab levels in BAL and plasma. BAL had much higher drug exposure than plasma. The BAL-to-plasma ratio (Figure 5A) of each animal showed that at initial time points, BAL levels were more than 100- to 1000-fold higher than plasma. By day 14, the BAL-to-plasma ratio was almost 1, showing similar levels in BAL and plasma. Figure 4(A2) shows trastuzumab concentration in the lungs, trachea, and bronchi. The levels in these tissues at the initial time points would depend on what part of the respiratory tract the dose was deposited while dosing. At later time points, higher levels were achieved in the lungs compared to the trachea and bronchi. This suggests that pulmonary dosing would result in higher levels in the peripheral lungs than in the upper and lower airways.

#### 3.3.2. F(ab′)2 PK

The PK of the F(ab′)2 fragment of trastuzumab after IV dosing is presented in Figure 3B. The *Cmax* for BAL and lung tissue was observed at 2 h, while it occurred at 10 min for the bronchi and trachea. After 12 h, the concentration of the drug in bronchoalveolar lavage (BAL) exceeded the concentration in the lungs, trachea, and bronchi. The elimination kinetics of F(ab′)2 in each tissue differed, indicating varying degradation/clearance rates in the tissues. F(ab′)2 demonstrated greater penetration in BAL and lungs compared to trastuzumab (refer to Table 1); however, in the trachea and bronchi, there was a reversal in trend with reduced penetration for F(ab′)2.

Figure 4(B1,B2) illustrate the PK of F(ab′)2 after pulmonary dosing. The observed F(ab′)2 concentrations in BAL were significantly higher than plasma levels, with plasma levels falling below the lower limit of quantification at multiple time points. The pulmonary PK of F(ab′)2 after pulmonary dosing generally indicated higher levels in lung tissue compared to the trachea and bronchi, consistent with the pattern observed for trastuzumab.

#### 3.3.3. Fab PK

Following systemic dosing, the PK analysis of the Fab fragment of trastuzumab (Figure 3C) indicated higher levels in bronchoalveolar lavage (BAL) and increased penetration into BAL with decreasing molecular size of the protein. The exposure of Fab in BAL was nearly 50% of that in plasma, which was significantly higher than in the lungs, trachea, and bronchi. The lungs, trachea, and bronchi reached maximum concentrations within 10 min, while the *Cmax* in BAL was observed at 6 h. The PK data suggest faster elimination of Fab fragments from the lung, trachea, and bronchi tissues compared to F(ab′)2 from the same tissues. Fab fragments exhibited a similar trend to trastuzumab and F(ab′)2 fragments in pulmonary tissues, with a higher *Cmax* in the bronchi, followed by the lungs and trachea.

In Figure 4(C1,C2), the PK of the Fab after pulmonary dosing is depicted. Similar to trastuzumab and F(ab′)2, the plasma levels of Fab were notably lower than BAL levels. The observed trends with the pulmonary exposure of Fab were also similar to those of trastuzumab and F(ab′)2, with the lungs showing the highest exposure, followed by the trachea and bronchi.

#### 3.3.4. scFv PK

In Figure 3D, the PK of scFv after systemic dosing is illustrated. The concentration of scFv in the bronchoalveolar lavage (BAL) exceeded the plasma concentration after 2 h. In the pulmonary tissues, scFv showed the highest concentration in the lungs, followed by the bronchi and trachea. This distribution pattern was opposite to what was observed with trastuzumab, F(ab′)2, and Fab, where the levels in the bronchi were higher than in the lungs. These data suggest that there is a size-specific disposition in pulmonary tissues.

Figure 4(D1,D2) show the PK profile of scFv after pulmonary dosing. Similar to trastuzumab and other fragments, the concentrations in bronchoalveolar lavage (BAL) were more than 10-fold higher than the concentrations in plasma. The PK in pulmonary tissues also followed a similar pattern to trastuzumab and other fragments, with the highest concentrations in the lungs, followed by the trachea and bronchi.

#### 3.3.5. Dose-Normalized PK of Protein Therapeutics

**Figure 6:**
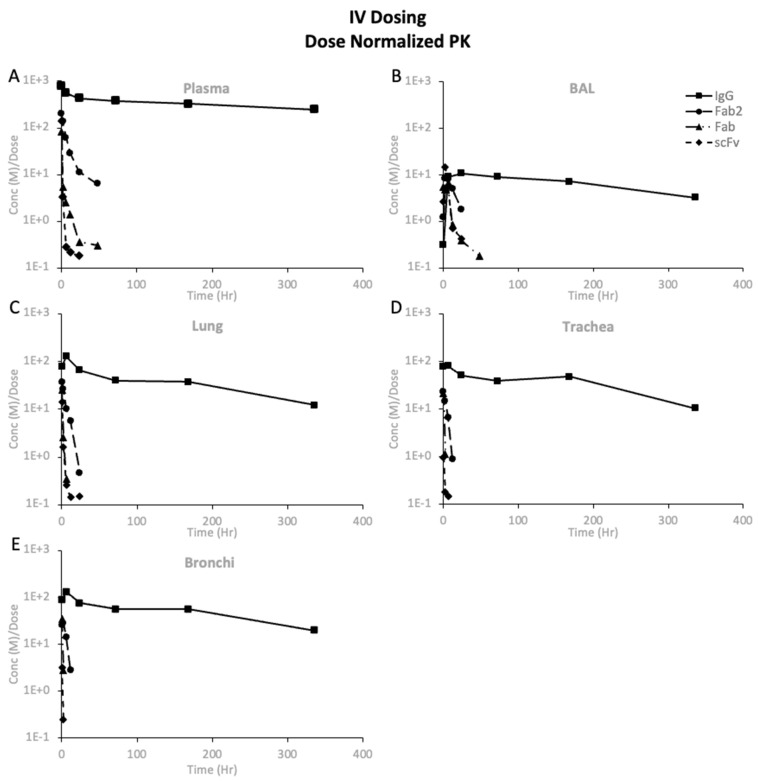
Dose-normalized PK (mean (n = 3)) after systemic dosing of proteins at 10 mg/kg dose: (**A**) plasma, (**B**) BAL, (**C**) lungs, (**D**) trachea, and (**E**) bronchi.

**Figure 7:**
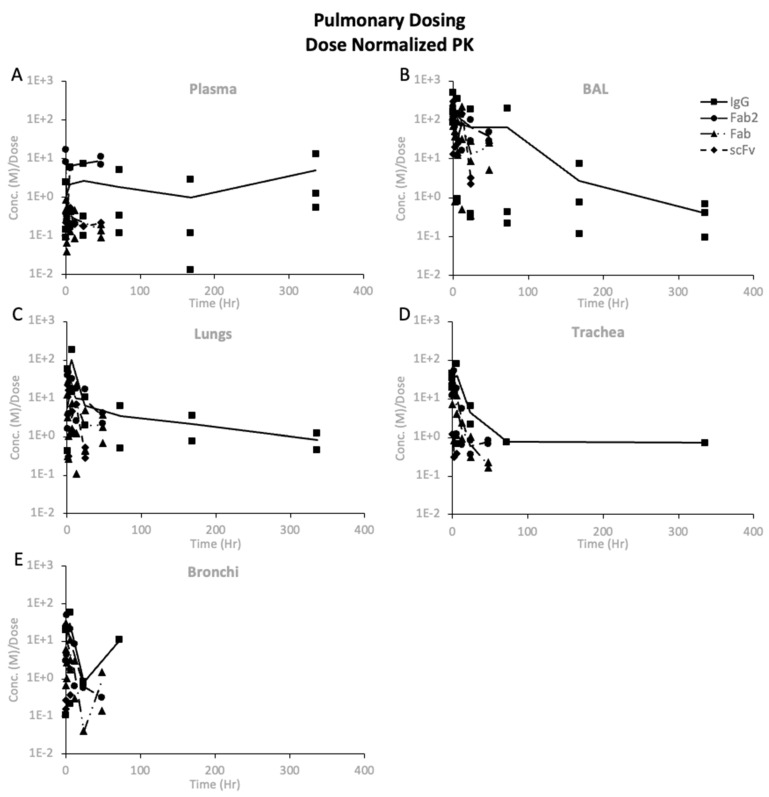
Dose-normalized protein PK in pulmonary tissues after intratracheal administration of 10 mg/kg dose: (**A**) plasma, (**B**) BAL, (**C**) lungs, (**D**) trachea, and (**E**) bronchi. Solid lines represent mean values, and symbols represent individual animals.

The dose-normalized PK showed differences in exposure after systemic and pulmonary dosing, as shown in Figure 6, Figure 7, and Figures S6 and S7. Comparing the PK of proteins after systemic dosing in Figure 6, one can see that all proteins’ maximum dose-normalized concentration in BAL is similar. However, *Cmax* decreased with decreasing size in the plasma, lungs, trachea, and bronchi. The dose-normalized PK after systemic dosing showed that the maximum dose-normalized concentration in BAL, lungs, trachea, and bronchi was consistent across proteins, except for scFv, where the trachea and bronchi exposures were lower. When comparing systemic and pulmonary dosing, we see that pulmonary dosing results in much higher BAL concentrations and much lower plasma concentrations than systemic dosing. The dose-normalized concentrations for the lungs, trachea, and bronchi were in a similar range following systemic and pulmonary dosing.

#### 3.3.6. Biodistribution Coefficients

**Figure 8:**
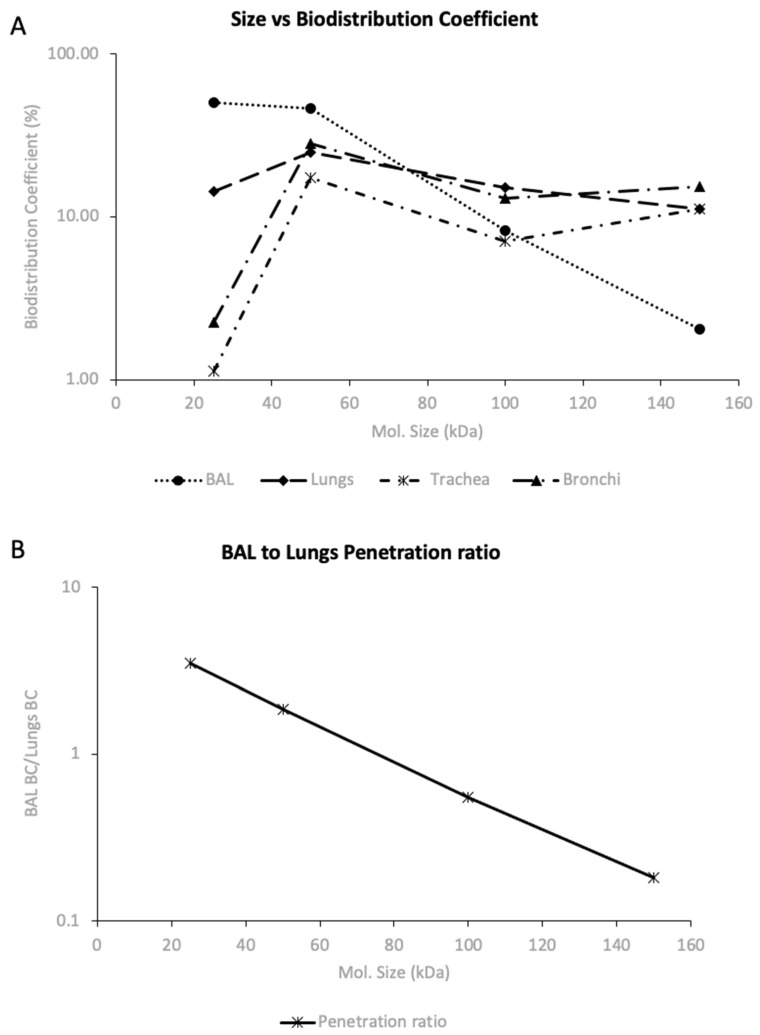
(**A**) Biodistribution coefficient (BC%) vs. size relationship for different proteins. BC% is a measure of tissue distribution determined using tissue and plasma AUCs; (**B**) the ratio of BAL and lung BC% (i.e., BAL-to-lung penetration ratio) plotted against protein size.

Table 1 shows the exposures and BC% for trastuzumab, F(ab′)2, Fab, and scFv after systemic dosing. The BC% quantifies the extent of tissue distribution for proteins in respective tissues. Specific trends were observed in the pulmonary disposition of these different molecular-sized proteins. The penetration in BAL increased from about 2% for trastuzumab to about 50% for scFv. It was also observed that penetration in the trachea was the lowest among all the pulmonary tissues. For trastuzumab, the penetration in the trachea was comparable to that in the lungs, and it dropped in comparison to the lungs for F(ab′)2, Fab, and scFv fragments. It was observed that penetration in pulmonary tissues was significantly higher for Fab when compared to other proteins, which could hint toward a sweet spot in terms of the molecular size of protein and pulmonary exposure. Figure 8A shows the correlation between the molecular size and biodistribution of proteins in different pulmonary tissues. We can see that penetration in the lungs, trachea, and bronchi is the highest for Fab, while scFv has the highest exposure in BAL. Figure 8B shows how the ratio of penetration in BAL to that in the lungs changes with the molecular size of the protein. This analysis gives insight into the transport of proteins from lung tissue to the epithelial lining fluid and size-dependent transport across the epithelial cell membrane.

## 4. Discussion

The respiratory system can be functionally separated into two zones, namely the conducting zone, which consists of the trachea, bronchi, and nasal cavity, and the respiratory area, which consists of alveolar sacs [28]. The conducting pathways connect alveolar sacs to the atmosphere, aiding air filtration and acting as channels for airflow [29]. Though the upper and lower airways do not participate in gas exchange [30], these airways play an essential role in respiration and providing immunity against respiratory infections [31]. The epithelial lining fluid in these pathways has a detectable concentration of proteins, including IgG antibodies [32]. Still, there needs to be more understanding of the process involved in transporting proteins across the epithelial membranes of these pulmonary tissues. It is possible to treat several diseases like pulmonary infections, asthma, cystic fibrosis, etc., by maintaining efficacious levels of therapeutic proteins in these pulmonary tissues. Thus, it is essential to investigate the determinants of protein biodistribution into these tissues.

Several in vitro and ex vivo investigations have explored the disposition of protein therapeutics across the alveolar epithelial cell layer [17,33,34]. These investigations demonstrate that the entry of IgG from the apical to the basolateral region is preferred over the reverse direction, and this preferential entry in the lung interstitium is mediated via FcRn [17]. IHC data in the literature show the presence of FcRn in human alveolar and bronchial epithelial cells. Thus, FcRn transcytosis could be the major process in transporting IgG from the apical to the basolateral membrane of epithelial cells. As such, one might be tempted to design protein therapeutics with the Fc domain (e.g., mAb or fusion protein like Epo-Fc) to accomplish high lung exposure following local administration. However, depending on the dose and bioavailability limitations, the extent of exposure may be similar to what is accomplished following systemic administration. In addition, if the goal is to achieve higher drug exposure in the epithelial lining fluid (ELF), one might want to design a therapeutic protein without a functional Fc domain. However, due to the local degradation of proteins in the lung ELF [35], it is unknown if one can accomplish significantly higher therapeutic exposure via this strategy compared to systemic administration. As such, there is a need to perform dedicated in vivo investigations to help understand the role of molecular properties (e.g., molecular size, FcRn binding, charge, etc.) and the route of administration on the pulmonary exposure of protein therapeutics. In this manuscript, we have presented an in vivo investigation that provides insights into the effect of molecular size (and indirectly FcRn binding) on the pulmonary disposition of protein therapeutics. Understanding the PK after the administration of trastuzumab, F(ab′)2, Fab, and scFv fragments of trastuzumab helped understand the effect of molecular size and comparing the PK of trastuzumab and F(ab′)2 gave insights into the role of FcRn binding.

Our investigation quantified protein concentrations in the lungs, trachea, bronchi, and BAL tissues using ELISA. It is important to note that the bronchi tissues collected comprise early bronchioles, not terminal bronchioles. Also, the volume of bronchial tissue in mice was small, less than 5 mg, allowing for only a singular ELISA analysis. With such small tissue volumes and low partitioning in tissue, it is crucial to have the correct dilution to capture the concentration range and reduce any matrix effect. Since the size of the trachea and bronchi in mice is small, it is challenging to have enough blank tissues to create a separate empty matrix for ELISA in these tissues. Consequently, the lung tissue matrix was used to quantify protein concentration in the trachea and bronchi using ELISA.

The proteins used in our in vivo study bind to human epidermal growth factor receptor 2 (HER2) and have no cross-reactivity in mice. Thus, these proteins do not exhibit target-mediated disposition and are ideal for studying molecular size’s effect on protein disposition in mice. Trastuzumab is a humanized mAb and has the potential to elicit immunogenicity in the clinic and preclinical species like mice. Although we did not look at anti-trastuzumab antibody levels in our study, an analysis of protein PK in plasma suggests that immunogenicity had a minimal effect on PK. Also, the literature supports that the potential for trastuzumab immunogenicity in mice is relatively low after a single administration [36].

After systemic administration, PK data in pulmonary tissues showed a correlation between molecular size and exposure. As the molecular size decreased, the exposure in the epithelial lining fluid increased. Protein entry from the lung tissue to the ELF can be through paracellular transport and pinocytosis (FcRn-mediated transcytosis for proteins having functional Fc portions). The ratio of penetration in BAL to penetration in lungs against molecular size (Figure 8B) suggests a clear trend in higher penetration from lungs to ELF with a decrease in molecular size. Thus, transport across the epithelial membrane may be size-dependent, and designing smaller-sized proteins would help achieve higher and faster levels of penetration into lung ELF following systemic administration. The molecular size vs. BC% plot shows a possible sweet spot for the molecular size of proteins in terms of penetration in pulmonary tissues. The Fab fragment, with a molecular size of 50 kDa, had the highest penetration in these tissues. Also, this trend hints at size-specific clearance mechanisms from ELF and other tissues, which may be mediated by immune cells. As the molecular size of the protein decreased, the penetration in ELF increased significantly, resulting in higher *Cmax* in ELF and lower systemic exposure for the proteins. Lower systemic exposure might be desirable to reduce any possible systemic toxicity arising from therapies targeting lung disorders.

In general, higher exposure and *Cmax* were observed in the bronchi, followed by the lung, and the lowest exposure was observed in the trachea. Still, for scFv, it was observed that the highest exposure was in the lungs, followed by the bronchi and trachea. Thus, there could be a rationale for designing proteins with a lower molecular weight to target peripheral lungs and for higher-molecular-weight species to target the lower airways. Trastuzumab PK in the trachea, bronchi, and ELF followed the same elimination kinetics as plasma PK. The detectable levels were observed in these tissues till day 14, suggesting the presence of FcRn-mediated transcytosis and recycling in these tissues. F(ab′)2 levels in the pulmonary tissues dropped quickly after reaching a *Cmax* and were not detectable after 12 h. This faster clearance of F(ab′)2 may be attributed to molecular-size-dependent degradation and the absence of FcRn recycling of F(ab′)2.

We saw highly variable PK in plasma and pulmonary tissues after pulmonary dosing; there were multiple time points where the protein concentrations dropped below the limit of quantification in tissues and plasma. This variability in the PK reflects the complexity involved with inhalation dosing. The inhalation of drugs is a complex process, and many determinants are responsible for different deposition patterns. The main mechanisms involved in deposition are inertial impaction, gravitational sedimentation, and Brownian diffusion. The relative contribution of these mechanisms toward deposition depends on the particle’s physical characteristics, the lungs’ structure, and breathing patterns. The particle size of aerosolized drugs is also an essential parameter for efficiently delivering drugs to different regions of the pulmonary systems. Usually, particles with size >5 µm tend to deposit in the upper airways, those 2–5 µm in size deposit in the lower airways, and those with size <2 µm deposit preferentially in the alveolar pathway [37].

It is not easy to control the breathing of mice while dosing proteins through a catheter. Thus, it is possible that when the introduction of proteins in the trachea coincided with inhalation, a greater fraction of aerosolized particles were deposited in peripheral lungs. There was no trend regarding higher lung levels resulting in higher plasma levels. In some instances, lower levels in the lungs and higher levels in the upper and lower airways translated to higher plasma levels. Thus, it is challenging to understand if there is a clear relation between the region of the airways where the drug was deposited and plasma levels. Also, alveolar macrophages and mucociliary pathways are known innate defense mechanisms of the body against all inhaled foreign objects. The variability observed in pulmonary PK could likely be a function of both variability in breathing patterns and variable innate clearance mechanisms of each animal [38]. Variability resulting from pulmonary dosing could potentially be reduced by using more sophisticated equipment specifically designed for inhalation dosing.

After pulmonary dosing, a clear trend was observed when comparing BAL to plasma levels for proteins, where BAL levels were significantly higher than plasma. The dose-normalized PK showed that BAL exposures were 10–100-fold higher after pulmonary dosing for the same dose of proteins compared to systemic dosing. Thus, this cements that inhalation is superior in quickly achieving higher levels of proteins in BAL than systemic dosing. The same cannot be concluded for protein concentration in the lungs, trachea, and bronchi since there was not much difference in exposure between systemic and pulmonary dosing. Pulmonary dosing resulted in higher lung concentrations than trachea and bronchi concentrations, unlike the systemic dosing of trastuzumab, F(ab′)2, and Fab. Thus, pulmonary dosing might be superior to systemic dosing if the target tissue is the peripheral lungs, not the airways.

The in vivo studies we conducted here to understand pulmonary PK after systemic and pulmonary dosing provide valuable and unprecedented insights into the differences in protein PK due to molecular size, FcRn binding, and the route of administration. Depending on the region of the pulmonary tissue the target lies in, one could design drugs with different molecular sizes and FcRn binding. However, more work must be done to understand the effect of FcRn binding more thoroughly on the pulmonary PK of proteins. Although pulmonary dosing seems to provide advantages over systemic dosing when targeting the epithelial lining fluid in pulmonary tissues, as our studies showed, controlling the fraction of drug delivered to peripheral lungs is very challenging, and more sophisticated methods should be used for pulmonary dosing. The most significant deterrent against choosing inhalation as a delivery route would be the variability in PK one might expect with this route of administration. As such, the data presented in this manuscript could be used as a guidance tool to develop next-generation protein therapeutics for increased pulmonary tissue exposure.

## 5. Conclusions

In conclusion, our findings suggest that the distribution of protein therapeutics to the lungs, upper airways, and lower airways after systemic administration is dependent on the size of proteins. A molecular size of about 50 kDa resulted in the highest exposure of protein therapeutics in the lungs, trachea, and bronchi, while 25 kDa protein resulted in the highest exposure in BAL. Our data also show that pulmonary dosing results in higher BAL-to-plasma ratios compared to systemic dosing, suggesting that local pulmonary dosing might be superior in attaining a higher exposure of protein therapeutics in the lung ELF.

## Figures and Tables

**Table 1:** *AUC*_0–*last*_ (M*hr) and *BC*% for trastuzumab, F(ab′)2, Fab and scFv fragments in lungs, trachea, bronchi, and BAL after systemic dosing.

	Trastuzumab		F(ab′)2		Fab		scFv	
Tissue	*AUC*_0–*last*_	*BC*%	*AUC*_0–*last*_	*BC*%	*AUC*_0–*last*_	*BC*%	*AUC*_0–*last*_	*BC*%
Plasma	2.13 × 10^−4^		4.08 × 10^−6^		8.05 × 10^−7^		1.89 × 10^−6^	
Lungs	2.38 × 10^−5^	11.17	6.18 × 10^−7^	15.13	1.99 × 10^−7^	24.74	2.70 × 10^−7^	14.32
Trachea	2.38 × 10^−5^	11.16	2.90 × 10^−7^	7.10	1.39 × 10^−7^	17.26	2.12 × 10^−8^	1.12
Bronchi	3.24 × 10^−5^	15.22	5.32 × 10^−7^	13.02	2.27 × 10^−7^	28.16	4.26 × 10^−8^	2.26
BAL	4.34 × 10^−6^	2.04	3.38 × 10^−7^	8.28	3.71 × 10^−7^	46.04	9.50 × 10^−7^	50.38

## Data Availability

The datasets generated in the current study are available from the corresponding author upon reasonable request.

## Supplementary Materials

The following supporting information can be downloaded at: https://www.mdpi.com/article/10.3390/pharmaceutics16101259/s1, Figure S1. Intratracheal intubation of mice under anesthesia; Figure S2. Lung tissue after dosing trypan blue via intratracheal instillation; Figure S3. SDS-PAGE analysis of purified F(ab′)2, Fab, and scFv; Figure S4. Representative ELISA standard curves; Figure S5. Urea assay standard curve; Figure S6. Dose-normalized PK after systemic administration; Figure S7. Dose-normalized protein PK after intratracheal administration.
