# Supplementary material for: Pulmonary Pharmacokinetics of Antibody and Antibody Fragments Following Systemic and Local Administration in Mice
Source: Pharmaceutics. 2024 Sep 27;16(10):1259. doi: 10.3390/pharmaceutics16101259 (PMC11510323; doi:10.3390/pharmaceutics16101259)
Supplement: Supplementary file 1 [file pharmaceutics-16-01259-s001.zip › pharmaceutics-3140579-supplementary.pdf]

Supplementary Material

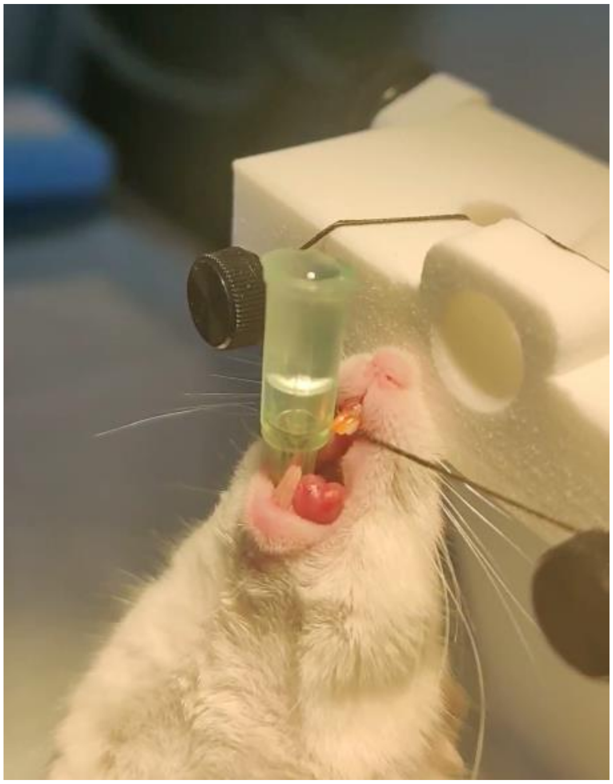

Figure S1. Intratracheal intubation of mice under anesthesia. The mice were intubated with a catheter, and the placement of the catheter in the trachea was confirmed either by looking for condensation around the catheter due to breathing or by adding 10 ul of PBS to the catheter (PBS would form pulses due to breathing).

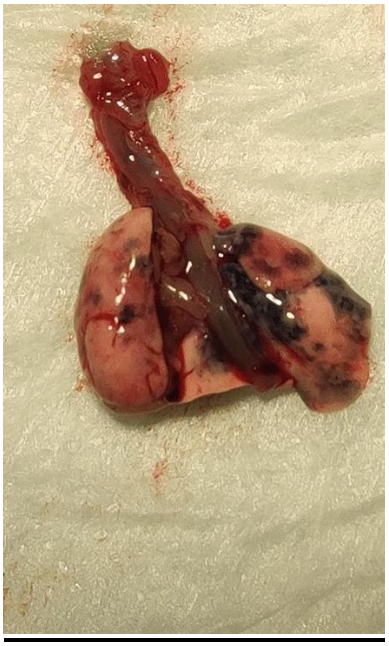

Figure S2. Lung tissue after dosing trypan blue via intratracheal instillation. Blue dye in the lungs confirms pulmonary delivery of trypan blue.

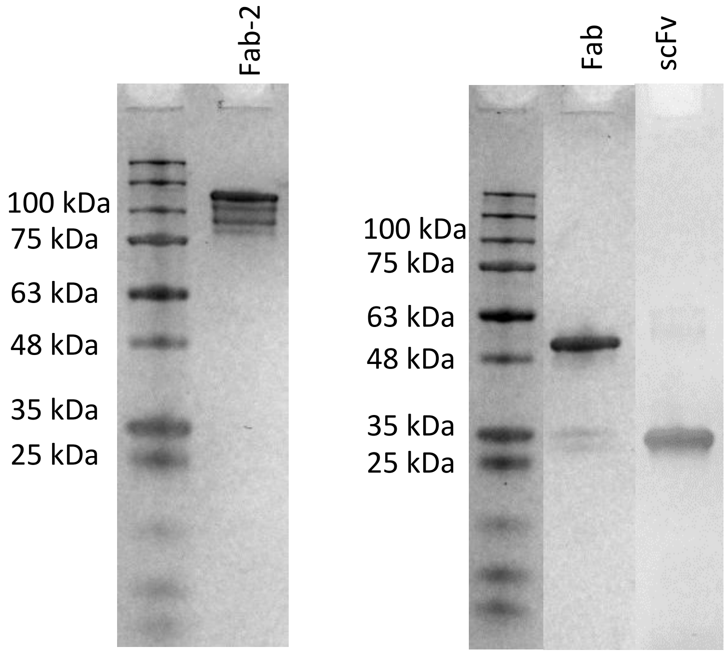

Figure S3. SDS PAGE analysis of purified F(ab)2, Fab, and scFv.

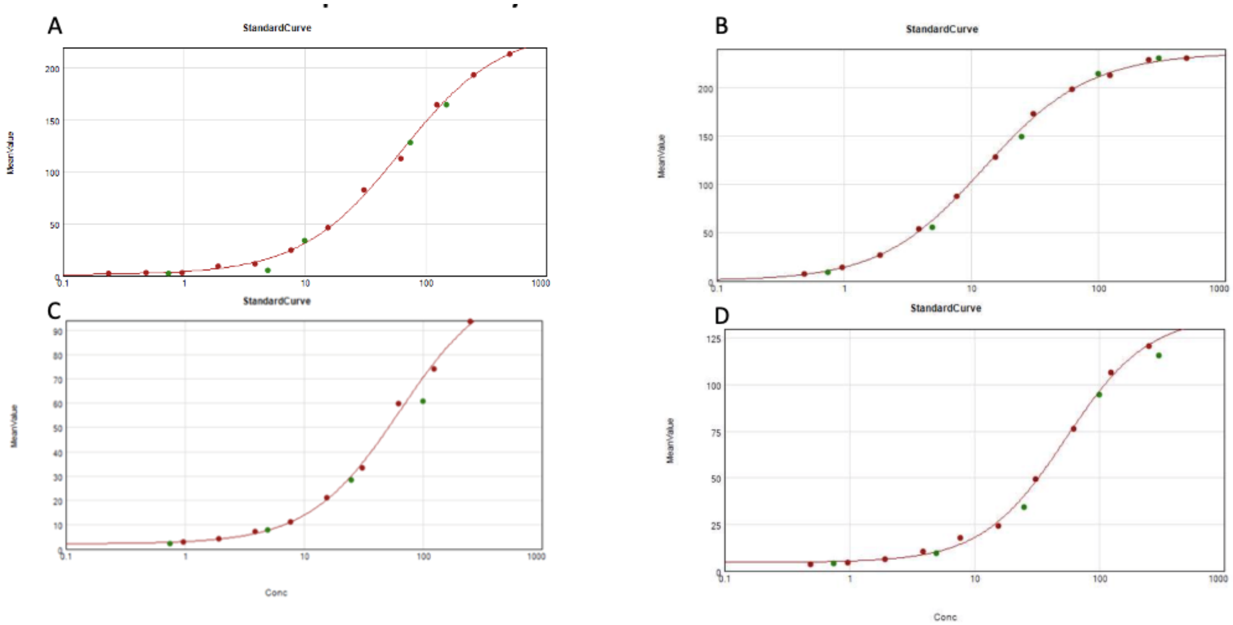

Figure S4. Representative ELISA Standard curves for trastuzumab, F(ab)2, Fab, and scFv fragments of Trastuzumab in Lung matrix. The solid red symbols are the standard readings, and the solid green symbols represent the QC readings.

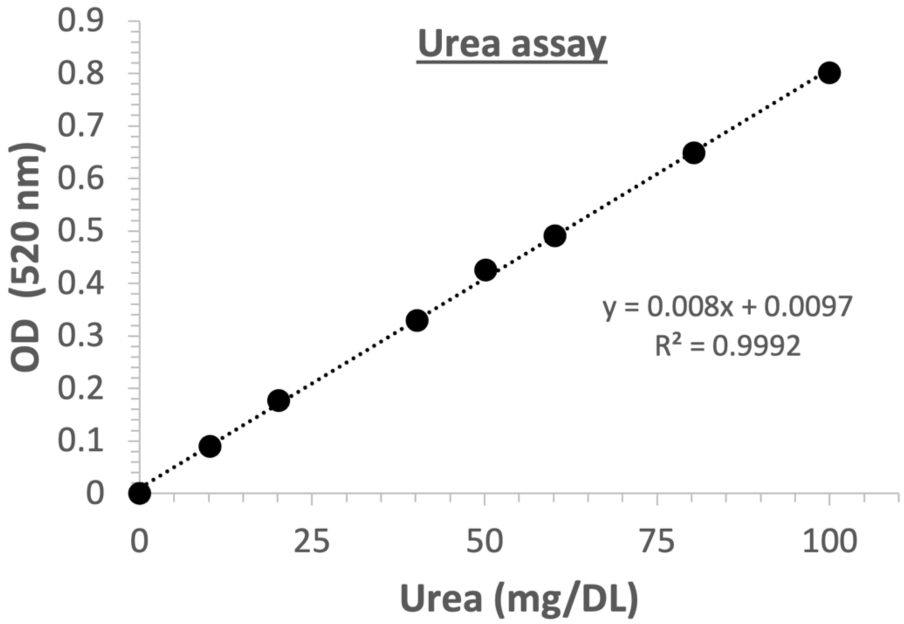

Figure S5. Urea Assay standard curve used to calculate urea concentrations in plasma and BAL samples.

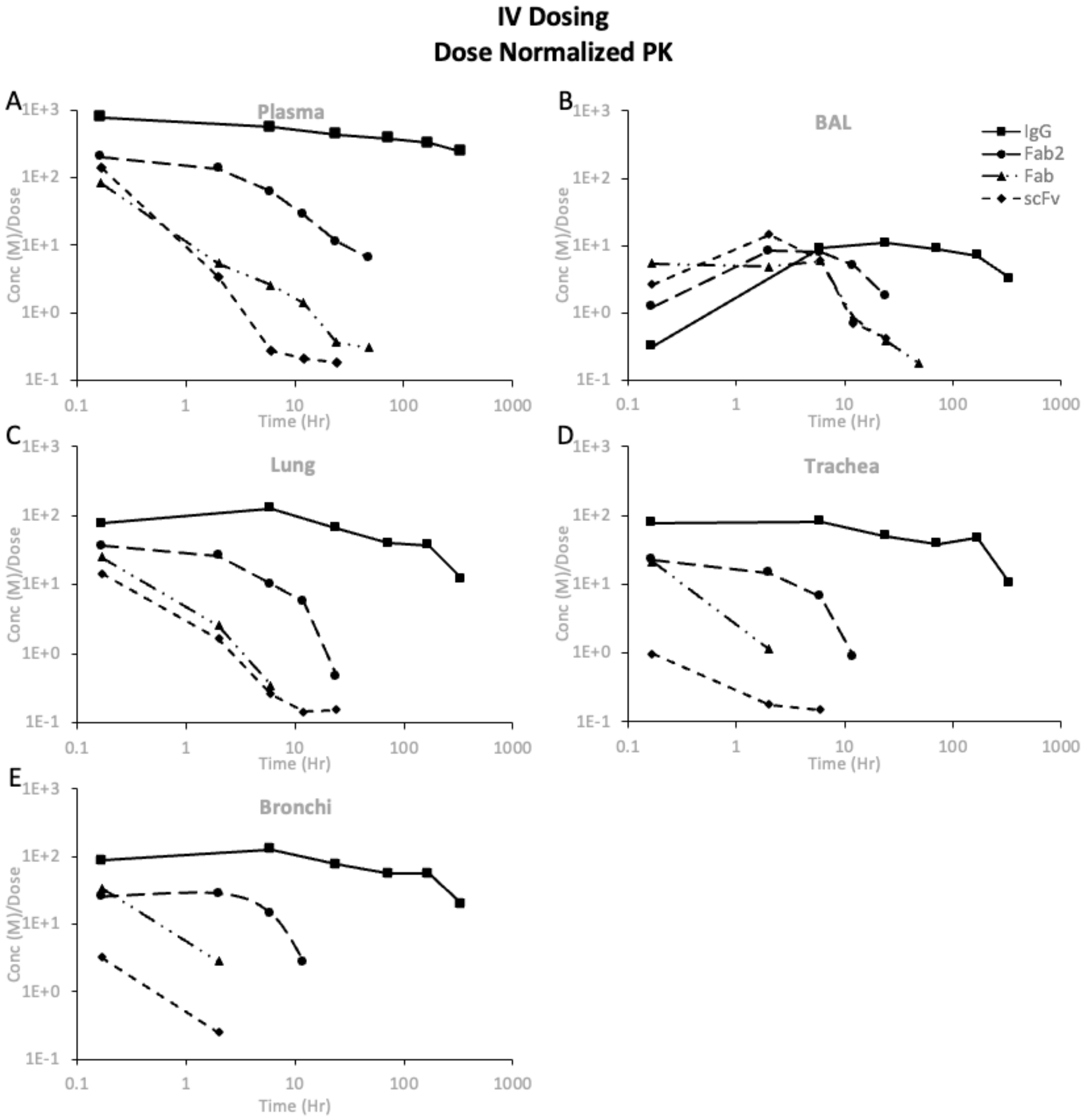

Figure S6. Dose normalized PK (mean (n=3)) after systemic dosing of proteins at 10 mg/kg dose (log-log scale). (A) Plasma, (B) BAL, (C) Lungs, (D) Trachea, and (E) Bronchi.

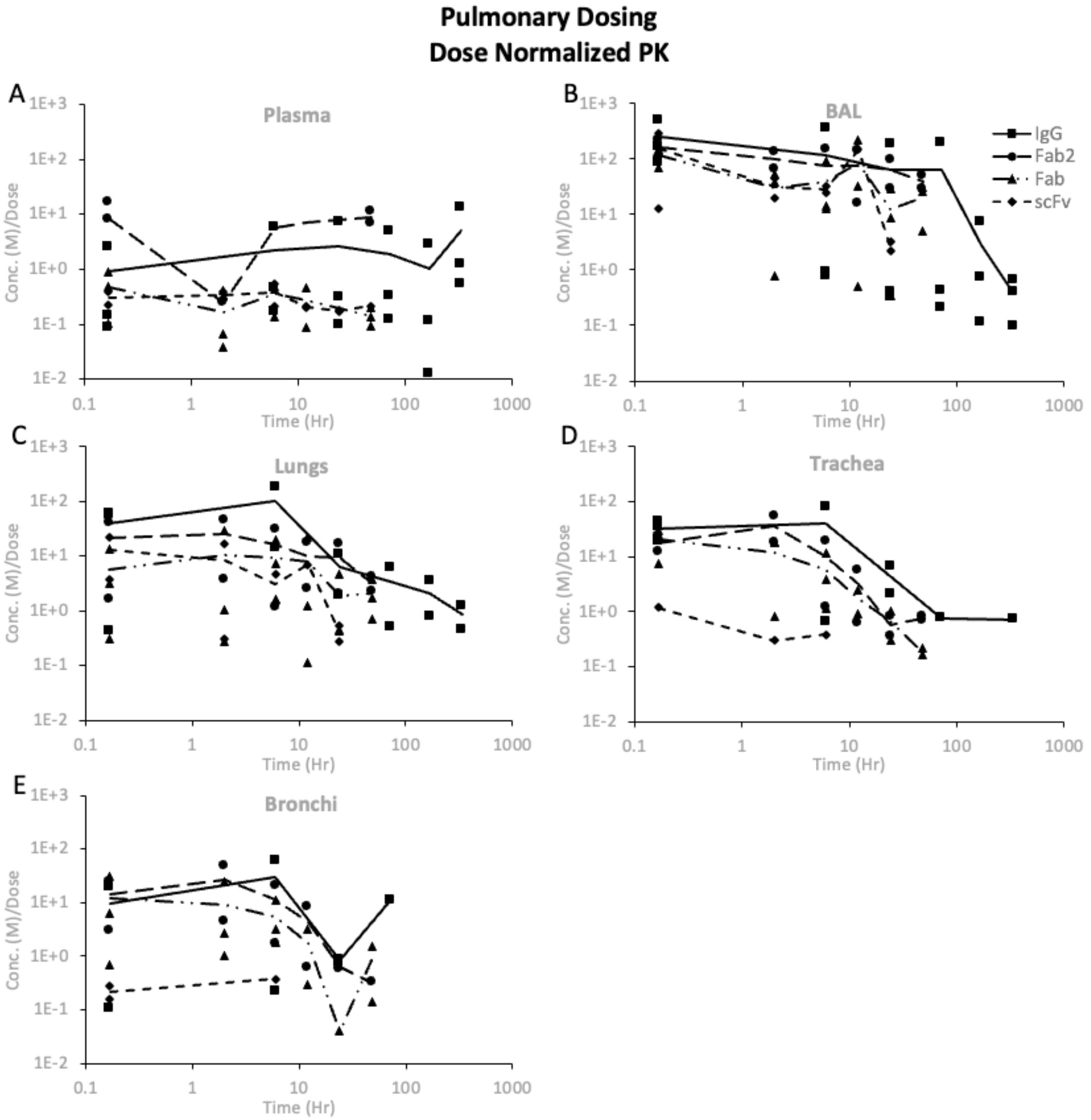

Figure S7. Dose Normalized protein PK in pulmonary tissues after intratracheal administration of 10 mg/kg dose (log-log scale). (A) Plasma, (B) BAL, (C) Lungs, (D) Trachea, and (E) Bronchi. Solid lines represent mean values, and symbols represent individual animals.
